# Radioprotective Effects of Combined Melatonin and Famotidine Treatment on Radiation Induced Apoptosis in Peripheral Blood Leukocytes of Breast Cancer Patients and Normal Individuals

**DOI:** 10.22074/cellj.2021.7378

**Published:** 2021-10-30

**Authors:** Elham Samei, Hossein Mozdarani, Farhad Samiei, Gholamreza Javadi

**Affiliations:** 1 Department of Genetics, Science and Research Branch, Islamic Azad University, Tehran, Iran; 2 Department of Medical Genetics, Faculty of Medical Sciences, Tarbiat Modares University, Tehran, Iran; 3 Department of Radiotherapy, Cancer Institute, Tehran University of Medical Sciences, Tehran, Iran

**Keywords:** Antioxidants, Apoptosis, Breast Cancer, Ionizing Radiation, Leukocytes

## Abstract

**Objective:**

The aim of this study was to evaluate the effects of individual or combined use of two antioxidants, melatonin and famotidine on radiation induced apoptosis in leukocytes from breast cancer (BC) patients.

**Materials and Methods:**

In this experimental study, the DPPH assay was used to determine the appropriate doses of melatonin and famotidine for treatment of BC and control leukocytes. The leukocytes were cultured in complete RPMI- 1640 medium and treated with either agent for two hours. Cells were exposed to 4 Gy gamma rays generated from a Co-60 source at a dose rate of 0.85 Gy for 48 hours before harvesting. The cells were placed on slides and the neutral comet assay was performed. A total of 500 cells were stained with ethidium bromide and assessed for the amount of apoptosis under a fluorescent microscope x400 magnification.

**Results:**

We observed significantly more apoptosis following radiation alone in the leukocytes from BC patients compared with normal individuals (P<0.01). Individual use of famotidine and melatonin induced very low frequencies of apoptosis that was not significantly different from the control (P>0.05). However, when combined with radiation, there was a decreased frequency of apoptosis in leukocytes of both normal and BC patients (P<0.05). The effect of famotidine was more pronounced than melatonin.

**Conclusion:**

Melatonin, despite its potent antioxidant property, does not significantly affect radiation induced apoptosis in leukocytes derived from normal individuals; however, it has a moderately significant protective effect on in leukocytes derived from BC patients. Therefore, when used with radiation it might not intervene with the radiotherapy (RT) regimen of BC cancer patients. Famotidine is a good radioprotector for normal tissue. However, the efficacy of RT might be reduced with an accumulation of famotidine in tumour tissues.

## Introduction

Breast cancer (BC) is one of the most common cancers and leading causes of death in women. The prevalence of BC in Iran is increasing and affected people are relatively younger compared to other countries (1, 2). About 80% of patients with BC receive radiotherapy (RT) that involves the use of ionizing radiation (IR). IR leads to cellular and molecular damages via direct or indirect actions. Therefore, chromosomal aberrations, cell death, alterations in the oxidation status of cells and alterations in cellular haemostasis in tumours as well as normal tissues are expected after irradiation (3). Prominent effects of sparsely IR such as X-rays or gamma rays include the formation of free radicals that interact with nucleic acids and lead to DNA damage. IR produces a variety of damages in DNA. From these, double-strand breaks are most critical effects that lead to chromosomal aberrations and two different modes of cell death termed mitotic or clonogenic cell death and apoptosis (4). Patients with BC show various biological reactions to RT that range from mild to acute adverse effects and include skin erythema, fibrosis, immunologic complications, or secondary cancers (5, 6).

The results of studies have shown that about 40% of patients with BC are sensitive to radiation (7-9). Therefore, it is of utmost importance to reduce the radiation side effects for these patients. To date, different naturally occurring or synthetic agents have been used to countermeasure radiation side effects. From various available agents, antioxidants such as melatonin and famotidine are reported to effectively reduce radiation induced cellular damages in normal tissues.

The results of studies show that H2 receptor antagonists such as cimetidine and famotidine, which are usually used to treat peptic ulcers, can be potent hydroxyl radical scavengers (10, 11). The radioprotective effects of these agents on radiation induced chromosomal aberrations and micronuclei in mouse bone marrow cells and human peripheral blood lymphocytes have been reported (12-14). Famotidine was shown to reduce radiation induced apoptosis in normal lymphocytes (15).

Melatonin, an indolic compound, is secreted at night by the pineal gland. Hardeland et al. (16) have published a review of the physiology and function of melatonin. Different studies have been performed to determine the oncostatic properties of melatonin against various tumours, including BC (17-19). Melatonin and its metabolites were found to be a direct free radical scavenger agent (20-22) that had the capability to stimulate the production of anti-oxidative enzymes and reduce the expression of pro-oxidative enzymes. Therefore, its use as a radioprotector and anti-cancer agent has been proposed (23). The anti-carcinogenic properties of melatonin and its anti-oxidative and free radical scavenging activity have been shown in different experimental models of carcinogenesis induced by oxidative damage inducing agents, which indicate the protective effects of melatonin (24-26).

The aim of this study was to evaluate the antiapoptotic effects of melatonin and famotidine alone or in combination on radiation induced apoptosis on lymphocytes of normal and BC individuals. BC patients have genomic instability (3); therefore, a different response to radiation in BC cells is expected compared to normal cells. To the best of our knowledge, there is no report about the combined treatment of famotidine and melatonin on radiation apoptosis induced in peripheral blood leukocytes of BC patients. Apoptosis was assessed by the neutral comet assay (single cell gel electrophoresis). The comet assay is reported to be a very reliable method for assessment of apoptosis induced by DNA damaging agents (15, 27, 28).

## Materials and Methods

### DPPH assay

In this experimental study, the DPPH assay, with 2, 2-diphenyl-1-picrylhydrazyl was used to evaluate the antioxidant properties of famotidine and melatonin in order to choose their optimum concentrations when combined with radiation. This method is an antioxidant assay based on electron-transfer that produces a violet solution in ethanol. This free radical, which is stable at room temperature, undergoes reduction in the presence of an antioxidant molecule and gives rise to a colourless ethanol solution. The DPPH assay was conducted according to standard procedure (29). The DPPH solution was prepared with 90% ethanol and we added various concentrations of melatonin and famotidine to this solution.After 30 minutes, the solution was read with an ELISA reader that had a 512 nm UV spectrum (BioTek, Taiwan). The percentage of absorbance was calculated using the following formula:


Inhibition=(OD control-OD sample)OD control×100%

### Blood sampling and drug treatment

The Ethical Committee at Natitional Institute for Medical Research Development (NIMAD, Tehran, Iran), approved this experimental study (IR.NIMAD.REC.1397.069). All participants gave written informed consent for study participation and completed a written questionnaire that asked information related to their life- styles. All non-smokers without viral infection, antibiotic consumption and X-ray at least one month prior to sample collection were included in the study. Table 1 lists the demographic information of the study participants. Venous blood samples were collected in heparinized vacutainers from 10 luminal A patients with BC whose age ranged between 23 and 66 years (mean: 37.4 ± 11) and 5 normal (control) individuals whose age ranged between 25 and 76 years (mean: 46 ± 13.9). Blood samples were divided into two parts: i. Not exposed to radiation-the control group that included untreated control, melatonin alone, famotidine alone, and combined melatonin-famotidine samples and ii. Exposed to gamma radiation, alone or in combination with famotidine and melatonin. Whole blood cultures were prepared by the addition of 0.1 ml blood to 0.4 ml RPMI-1640 medium (Gibco, BRL, UK) supplemented with antibiotics (penicillin 100 IU/ml and streptomycin 100 µg/ml, Sigma, USA), 10% L-glutamine (2 mM, Sigma, USA) and 15% foetal bovine serum (FBS, Gibco BRL, UK). Famotidine and melatonin powder (Chemodaru Pharmaceuticals, Iran) were dissolved in RPMI medium, then added to culture vessels two hours prior to irradiation at concentrations of 80 µg/ml (famotidine) and 800 µg/ml (melatonin).

**Table 1:** Study participants’ demographic information


Normal control					Mean age ± SD					
# 5					37.4 ± 11					
Luminal A	Age (Y)	Age at onset (Y)	R/L	Type	Grade	Stage	ER	PR	Her2	Ki67
BC patients										

P1	47	46	R	D	2	1A	70	70	N	25
P2	47	46	L	D	2	2A	100	100	N	25
P3	45	44	R	L	2	2A	80	80	N	12.5
P4	26	26	R	D	2	3A	100	100	N	13.5
P5	76	75	L	D	2	2B	100	100	N	11.5
P6	42	41	L	D	1	2A	100	90	N	7.5
P7	42	41	R	D	1	2A	90	90	N	5
P8	44	43	R	D	1	2A	100	90	N	13.5
P9	45	44	R	D	1	1A	95	70	N	2
P10	46	45	R	D	2	1A	100	100	N	4.5
Mean ± SD	46 ± 11.58	45.1 ± 11.41					93.5 ± 10.01	89 ± 11.36		12 ± 7.54


BC; Breast cancer, P1-P10; Patient number, R/L; Right/left, ER; Oestrogen receptor, PR; Progesterone receptor, and SD; Standard deviation.

### Irradiation

The culture vessels were irradiated with a therapeutic Co-60 gamma ray source (Theratrone, 780-C, Canada) at a dose of 4 Gy. The dose rate was 0.8 Gy/minute at a source to sample distance (SSD) of 80 cm. Irradiation was done at an ambient temperature (23 ± 2˚C). After irradiation, the cells were incubated at 37˚C for up to 48 hours.

### Neutral comet assay

**Fig.1:**
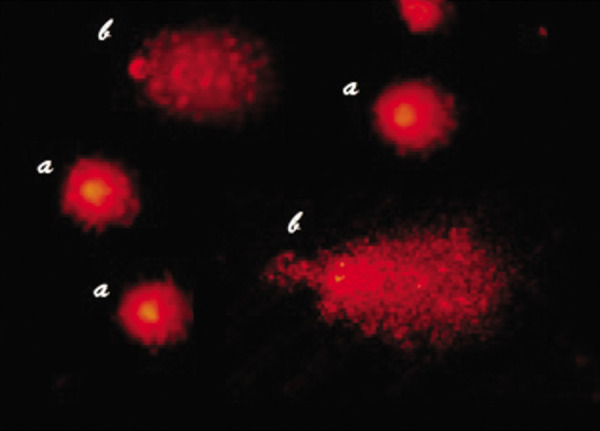
Typical photomicrographs of non-apoptotic and apoptotic neutral comet assay results. Apoptotic cells show a very small head and a fan-like tail. a; Non-apoptotic and b; Apoptotic (magnification: x400).

The neutral comet assay was used to assess apoptotic and non-apoptotic cells according to previously published protocols (15, 30) with minor modifications. Briefly, the previously incubated cells were centrifuged and the cell pellets were mixed with 0.75% low melting agarose (LMP, Fermentas, Germany) in phosphate-buffered saline (PBS) and immediately covered with a coverslip. The slides were kept at 4˚C for 15 minutes. After removal of the coverslips, the slides were transferred to lysis buffer that contained 2.5 M NaCl, 0.1 M EDTA, 10 mM Tris base, 1% N-lauryl sarcosine, 1% Triton X-100, and 10% dimethyl sulphoxide (DMSO, all from Merck, Germany) with a final pH of approximately 10. The slides were kept at 4˚C in the dark for 30 minutes, then washed with an electrophoresis buffer. After lysis, the slides were placed in a horizontal electrophoresis chamber that was filled with fresh electrophoresis buffer. Electrophoresis was conducted at 20 Volts and 100 mA. The slides were washed with distilled water for 5 minutes and then fixed in ethanol for 5 minutes at room temperature. The air-dried slides were stained with an ethidium bromide solution (20 μg/ml) and covered with coverslips. The number of apoptotic and non-apoptotic cells were scored using a fluorescent microscope (Nikon) equipped with an excitation filter (510-550 nm) and barrier filter (590 nm) at 400x magnification. Figure 1 shows typical normal and apoptotic cells analysed under the microscope. A total number of 500 cells were randomly assessed for each slide. 

### Statistical analysis

Data were analysed using SPSS software (version 18, SPSS Inc., USA). All data were first tested by using the Kolmogorov Smirnov test for normal distribution. Then, to compare the two groups, we used the Mann Whitney non-parametric test and analysis of variance (ANOVA) to compare more than two groups. P<0.05 were considered to be statistically significant. All figures were drawn with the use of GraphPad Prism software, version 4.0 (California Corporation, USA).

## Results

### DPPH assay

### Famotidine

**Fig.2:**
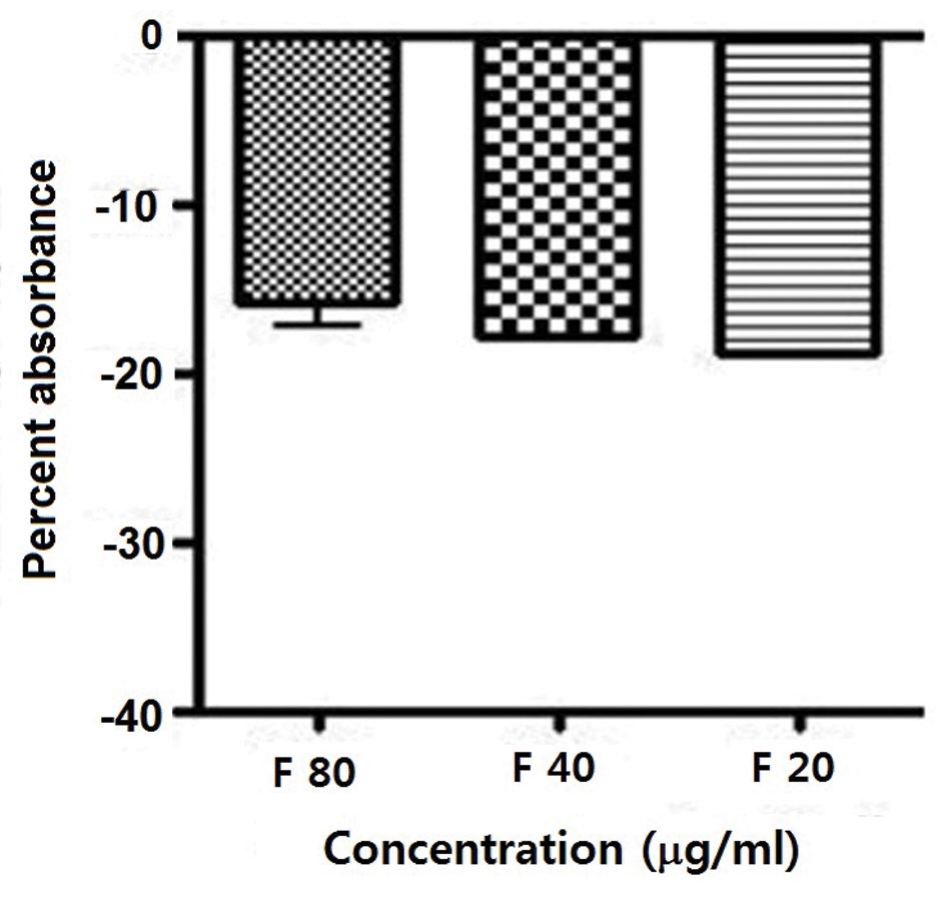
Percent absorbance of famotidine (F) as assayed with 2, 2-diphenyl-1- picrylhydrazyl (DPPH) and read by an ELISA reader with a 512 nm UV spectrum. Error bars show standard deviation (SD) of mean values from triplicate repeats.

As seen in Figure 2, famotidine did not show any antioxidant capacity. The higher dose of famotidine was more effective. There was no significant difference between the 20 µg/ml and 40 µg/ml concentrations (P>0.05). However, there was a statistically significant difference between the other doses and the 80 µg/ml dose (P<0.05). Therefore, we used the 80 µg/ml dose for all of the radiation experiments.

### Melatonin

**Fig.3:**
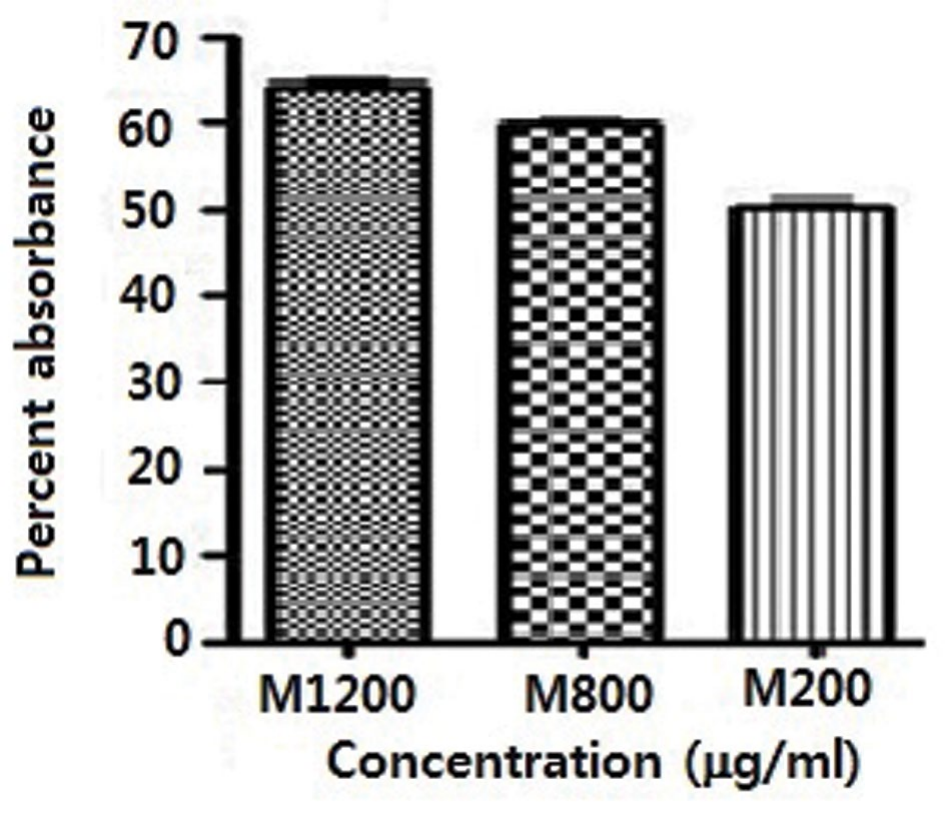
Percent absorbance of melatonin (M) as assayed with 2, 2-diphenyl-1-picrylhydrazyl (DPPH) and read by an ELISA reader with a 512 nm UV spectrum. Error bars show standard deviation (SD) of mean values from triplicate repeats.

Figure 3 shows the results of the DPPH assay for melatonin. There was a dose-dependent potent antioxidant capacity with melatonin. A significant difference existed between the 200 µg/ml dose and the 800 and 1200 µg/ ml doses (P<0.05). However, the difference between the 800 and 1200 µg/ml concentrations was not statistically significant (P>0.05). Therefore, we used the 800 µg/ml dose for all of the radiation experiments.

### Neutral comet assay

### Normal individuals

**Fig.4:**
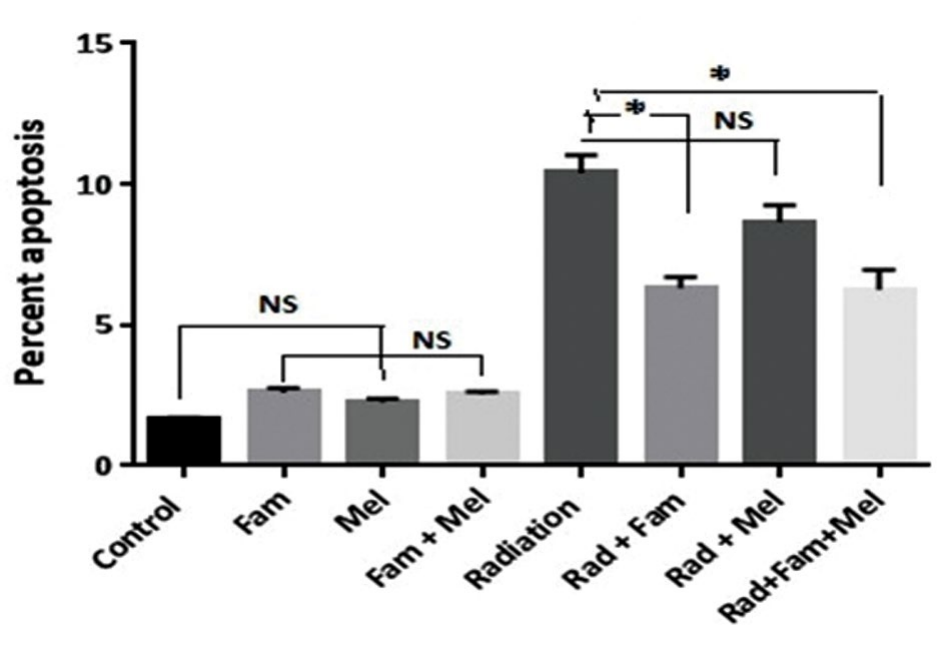
Treatment with famotidine (Fam) and melatonin (Mel), alone or in combination, prior to gamma irradiation of leukocytes from normal individuals. NS; Non-significant and *; P<0.01 error bars indicate standard error of mean (SEM).

Figure 4 shows the results of this assay. As seen, irradiation of whole blood leukocytes with gamma rays induced a comparatively high percentage of apoptosis compared to the control non-irradiated samples (P<0.01). There were no significant differences between the drug treatments whether used alone or in combination with the control group (P>0.05). There was no significant difference between radiation alone and in combination with melatonin (P>0.05). However, there were significant differences between radiation and famotidine alone and between famotidine in combination with melatonin (P<0.05).

### Breast cancer patients

**Fig.5:**
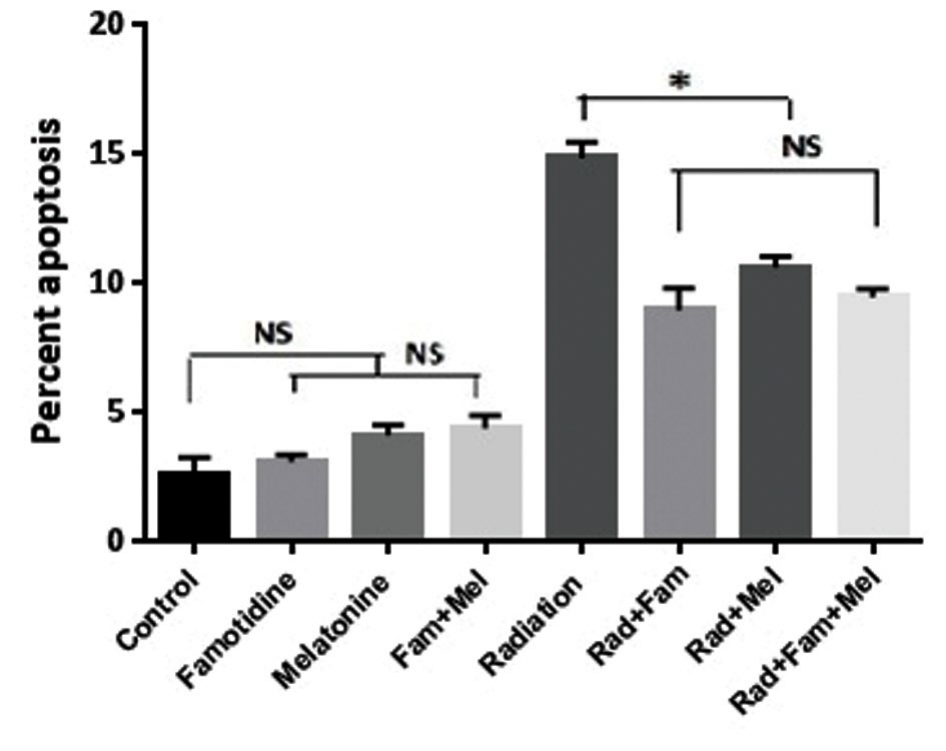
Treatment with famotidine and melatonin, alone or in combination, prior to gamma irradiation of leukocytes from breast cancer (BC) patients. NS; Non-significant, *; P<0.01 error bars indicate standard error of mean (SEM).

Figure 5 shows significant differences between the radiation and drug treatment groups (famotidine and melatonin), either alone or in combination (P<0.05). A higher background frequency of apoptosis was seen in the BC leukocytes (P<0.05). Radiation induced significantly higher frequency of apoptosis in leukocytes from BC patients compared to normal individuals (P<0.05).

A protective effect for radiation induced apoptosis was seen for both normal and BC leukocytes when radiation was combined with famotidine and famotidine plus melatonin (P<0.05). The results indicated no significant protective effect with melatonin combined with radiation in leukocytes from normal individuals (P>0.05); however, the effect was statistically significant for BC patients (P<0.05).

The comet assay results showed a significant increase in apoptosis following irradiation and significant decrease in the presence of combined melatonin and famotidine.

## Discussion

RT is an efficient treatment modality for about 50% of patients with malignant breast tumours. The direct and indirect effects of IR potent inducers of DNA damage, chromosomal instability and cell death in tumour and normal tissues. Most patients can tolerate RT; however, some suffer from severe adverse effects. This variability in response may be caused by a genetic predisposition and inherent radiosensitivity in BC patients (3, 31). The cytotoxic reactions of normal tissues to IR limits the efficiency of RT. Unfortunately, an appropriate protocol to prevent or treat these side effects has not been developed. Therefore, the inherent radiosensitivity of normal cells might be considered a serious problem in management of RT for BC. The use of radioprotectors has been proposed to reduce normal tissue radiotoxicity. To date, no appropriate single radioprotector has been introduced for this purpose. The combined regimen of chemical or naturally occurring antioxidants might be useful for BC patients. The application of famotidine and melatonin two hours prior to irradiation led to a significant reduction in the frequency of radiation induced apoptosis. A similar observation was made with leukocytes from BC patients, although there was a higher frequency of apoptosis. The combination of famotidine and melatonin was more effective than melatonin alone, but not as effective as famotidine alone. The DPPH assay results and results from other studies show that melatonin is potent antioxidant (32, 33). The radioprotective potential of melatonin is shown by different investigators using different end points such as protection of lymphocytes against gamma rays, reducing frequency of chromosomal aberration and micronuclei, and reducing radiation induced cytotoxicity in normal tissue (24-26, 34).

Melatonin might reduce DNA damage, because of its direct radical scavenging actions of free radicals induced by IR (35). Melatonin and most of its metabolites have the capability to scavenge free radicals and reactive nitrogen species (20). Moreover, melatonin stimulates the activities of antioxidant enzymes to remove ROS before damaging DNA and assists the mechanisms involved in DNA damage repair (36). Therefore, melatonin, as a potent antioxidant, exerts a radioprotective effect. Furthermore, besides being a potent antioxidant, melatonin is a potent inducer of apoptosis. It was shown that melatonin increased frequency of the programmed cell death induced by ROS generated by arsenic trioxide, activation of the p38/JNK pathways, and by upregulation of Redd1 expression in human BC cells (37).

The synergistic effect of melatonin has been shown with anti-cancer drugs, which led to effective anti-proliferative and pro-apoptotic activities in colon cancer cell lines by activating the cytochrome c/caspase signalling pathways (38). These observations might explain why the radioprotective effects of melatonin on normal lymphocytes did not significantly differ with radiation alone. Melatonin has been shown to enhance the radiosensitivity of cancer cellsthrough inhibition of proliferation, promotion of cell cycle arrest, and inhibition of proteins involved in DNA double-strand break repair (39).

Famotidine led to a considerable decrease in the frequency of gamma irradiation induced apoptosis. The results of previous studies showed the radioprotective potency of famotidine against gamma ray induced chromosomal and micronuclei induction (12, 14) as well as radiation induced apoptosis in normal cells (15). Ching et al. (10) previously reported that antagonists of the histamine H2 receptor such as cimetidine, famotidine and ranitidine are not only good inhibitors of histamine-stimulated gastric acid secretion, but also are potent radical scavengers. Although the antioxidant potency of famotidine has not been assessed as much as for melatonin, the reduction in frequency of radiation induced apoptosis by famotidine is much more considerable compared to melatonin. This observation is consistent with findings from other H2 receptor antagonists, cimetidine alone or in combination with famotidine, on gamma ray induced micronuclei in mouse bone marrow (13, 40). Famotidine is effective against radiation induced apoptosis via OH radical scavenging and an intracellular antioxidant mechanism (15). The combination of famotidine and melatonin used with radiation led to a protective effect that was similar to famotidine alone. The mechanism by which famotidine reduces radiation induced apoptosis is not clearly understood, but it may be due to its antioxidant properties and is not measurable by the DPPH assay.

## Conclusion

The results imply that melatonin, despite its potent antioxidant property, does not significantly affect radiation induced apoptosis in leukocytes derived from normal individuals. However, a moderate significant protection is induced in leukocytes derived from BC patients. When used with radiation, it might not intervene with the RT regimen for BC cancer patients. Famotidine, on the other hand is a good radioprotector for normal tissue, but if it accumulates in tumour tissues, it might reduce the efficacy of RT.
